# Race, Obesity, and Mental Health Among Older Adults in the United States: A Literature Review

**DOI:** 10.1093/geroni/igaa031

**Published:** 2020-08-01

**Authors:** Karen D Lincoln

**Affiliations:** Suzanne Dworak-Peck School of Social Work, University of Southern California, Los Angeles

**Keywords:** Body mass index, Mental health disorders, Overweight, Population heterogeneity, Racial/ethnic minority older adults

## Abstract

Rising rates of obesity among older adults in the United States are a serious public health concern. While the physical health consequences of obesity are well documented, the mental health consequences are less understood. This is especially the case among older adults in general and among racial and ethnic minority older adults in particular. Available studies document a link between obesity and a variety of mental health disorders. However, findings from this body of evidence are inconsistent, especially when race and ethnicity are considered. This article examines research on obesity and mental health among older adults and identifies risk factors, causal mechanisms, and methodological approaches that help clarify the equivocal nature of the literature. Promising research and future directions include studies that consider a wide array of contextual factors and population heterogeneity.

**Translational Significance:** Understanding the mechanisms that link obesity and mental health disorders among older adults can enhance efforts to identify high-risk older adults, improve targeted prevention and treatment, and increase the quality of life for older adults and their caregivers.

Despite public health efforts, obesity is now considered an epidemic in the United States and rising rates are expected to continue for decades. The increasing prevalence of obesity in the older adult population in general, and among older racial and ethnic minority adults in particular, is a serious public health concern and poses significant consequences for people across the life span. This phenomenon of an overweight older adult population is the source of much research and debate with regard to treatment recommendations, appropriate care, health care costs, and the physical health consequences for older adults. Fewer studies, however, consider the mental health consequences of being overweight. Consequently, the impact of being overweight on the mental health of older adults is generally unknown.

Because obesity is pervasive and persistent, it is important to understand the effects of it on all aspects of well-being. Available evidence suggests several broad conclusions about the relationship between obesity and mental health, the most important being that there is a causal link between these two conditions. In general, the body of evidence indicates that weight is associated with a range of mental health disorders, including depression (1,2), posttraumatic stress disorder (3), anxiety (4), and cognitive impairment (5). However, studies that report no association (6) and even a negative relationship between obesity and mental health disorders (7,8) raise questions about the reliability and generalizability of the findings for older adults in general and for racial and ethnic minority older adults in particular.

Studies of obesity and mental health among adults in later life are fairly underdeveloped. Consequently, knowledge of the mechanisms that link these two conditions in older adults is largely unknown. The lack of evidence in this area is especially concerning given the rising number of overweight and obese older adults. Unlike younger populations, older adults are more likely to have multiple chronic health conditions that increase their risk for mental health disorders such as depression. Being overweight or obese significantly complicates these conditions, resulting in greater disability for older adults, increased caregiver burden, and higher cost of care. Not only is the existing body of evidence not extensive enough, but it is also not inclusive enough to fully appreciate the implications of being overweight or obese and its impact on the overall well-being of older adults. More specifically, conclusions from extant studies of obesity and mental health are limited to the extent that they focus on older adult populations overall and diverse racial and ethnic groups specifically. This is an important omission given the higher prevalence of obesity in some racial and ethnic minority groups compared to whites (9).

A growing body of evidence suggests that the correlates of obesity may depend on race, ethnicity, and culture (10,11), which may determine the extent of vulnerability and susceptibility to obesity, as well as alter the link between physical health conditions and mental health problems (12–15). Studies that examine obesity and mental health among racial and ethnic minority groups demonstrate an interesting divergence from findings reported for the general population. For example, findings that overweight African American older adults (16) and underweight Korean older adults (17) have a lower risk for psychiatric disorders than whites are intriguing and generally unexplained in this literature. Moreover, studies that identify overweight and obesity as a protective factor for the mental health of African Americans and Asians, but not for whites or Hispanics (18–20), raise questions about our current understanding of the causal mechanisms that link weight to mental health disorders in diverse populations.

The purpose of this article is to review studies of obesity and mental health to better understand the relationship between these two conditions among older racial and ethnic minority populations. Based on the body of evidence, there are several competing conclusions that can be reached from current findings. First, one can accept that existing studies are methodologically and conceptually sound, and therefore conclude that obesity is a risk factor for mental health among racially and ethnically diverse older adults. I reject this conclusion based on limitations in a large portion of this research that employs samples that lack racial, ethnic, and age diversity, robust measures of obesity and mental health disorders, and study designs that account for change over time. Second, one could reject existing evidence and point to the equivocal nature of the findings and the lack of attention to age, race, and ethnicity and propose that more research is required before any conclusions can be reached. I also reject this conclusion and believe that the extant literature does contribute to knowledge about important risk and protective factors. A third conclusion, which forms the framework for this article, is that the disparate findings in the literature reflect the nature of populations—that is, there is wide variation within and between groups of people, including those who are obese, those with mental health disorders, and those who are members of a particular racial or ethnic group. This heterogeneity is reflected in findings that are seemingly equivocal, but are more likely reflecting some of the unmeasured variations within and between groups and over time that cannot be observed when using a comparative, cross-sectional approach. To understand the link between obesity and mental health in later life, it is important to consider population heterogeneity as well as a broad array of contextual factors that might explain the wide variation in findings observed in reported associations between obesity and mental health in older adults across race and ethnicity.

This article begins with an overview of the prevalence of obesity and mental health disorders in older adults. This discussion is followed by a summary of studies that examine the association between obesity and mental health in the general population of older adults. Next, the body of evidence that focuses on racial and ethnic minority groups will be described. Finally, studies that represent promising areas of research will be considered for their potential to explain variation in reported findings in the link between obesity and mental health across racial and ethnic groups.

## Obesity in the Adult Population

Currently, about 40% of adults in the United States are obese as defined by a body mass index (BMI) of greater or equal to 30 and about 20% of adults are severely obese as defined by a BMI of greater or equal to 40 (9). According to new projections published in the *New England Journal of Medicine*, nearly half (48.9%) of all adults in the United States will be obese by 2030 and nearly one quarter (24.2%) will be severely obese (21). As the proportion of the population older than age 65 grows and becomes more racially and ethnically diverse, so too increases the prevalence of those individuals who are overweight and obese. Although overweight and obesity rates have increased for Americans overall in the last decade (21), the largest increase has occurred among the older population and among racial and ethnic minority adults (9).

According to the Centers for Disease Control and Prevention, the prevalence of obesity is higher among middle-aged (42.8%) and older adults (41%) than among younger adults (35.7%). With respect to race and ethnicity, the overall prevalence of obesity is higher among non-Hispanic black (46.8%) and Hispanic adults (46%) than among non-Hispanic white (37.9%) and Asian adults (12.7%). African American women have the highest prevalence of obesity (54.8%), followed by Hispanic women (50.6%) and Hispanic men (43.1%), respectively (9). Severe obesity is more prevalent among women, black Americans, and low-income adults (21).

## Mental Health Among Older Adults

Overall, the prevalence of mental health disorders declines in older age (22,23). Despite declining rates, obesity can increase the risk of mental health disorders in this population. Studies show that overweight and obesity are associated with major depression, bipolar disorder, panic disorder (4), other anxiety disorders (24), posttraumatic stress disorder (3), personality disorders, eating disorders, and alcohol use disorder (24,25). Thus, the mental health of older adults cannot be fully appreciated without considering the current and projected rise in obesity among this population over the next decade.

Obesity and mental health are two different conditions that are typically examined in separate literatures, with few reporting prevalence rates for the co-occurrence of these conditions. However, available evidence on depression provides an interesting picture of the relationship between obesity and mental health across race and ethnicity. Key findings from the National Health and Nutrition Examination Surveys, 2005–2010 (the most recent statistics available) reported that 43% of adults with depression were obese, and adults with depression were more likely to be obese than adults without depression (26). The relationship between obesity and depression varied by race and ethnicity but only among women. Forty-five percent of white women with depression were obese, whereas 32% of white women who did not have depression were obese. However, among non-Hispanic black and Mexican American men and women, rates of obesity did not differ by depression status. These findings provide some evidence for the varying effects of race and ethnicity on the association between obesity and mental health and highlight the need for studies that attempt to explain this variation.

## Race and Ethnic Variation in Obesity and Mental Health

Despite the clear connection between obesity and mental health in the general population of older adults, findings are divergent when race and ethnicity are examined. Some studies report a higher risk for psychiatric disorders for overweight or obese white older adults compared to other racial and ethnic groups (27–30). For example, Xiang and An (31) used data from the Health and Retirement Study—a nationally representative longitudinal sample—to examine the onset of depressive symptoms among 6514 middle-aged and older adults. Findings indicated that being overweight was significantly associated with future onset of depressive symptoms among whites, but there was no association between weight and depression onset among African Americans or Hispanics. Other studies report a higher risk for depression among overweight or obese African Americans than whites (16). In a study of 2406 community-dwelling African American and white older adults, higher BMI was associated with higher levels of depressive symptoms 3 years later among African Americans overall, and in particular, those with lower levels of education. This relationship was not found among whites. In contrast, a study of Korean older adults found a significant association between weight and depression, but the findings diverged from those reported for African Americans and whites. Specifically, in a longitudinal representative sample of more than 10,000 Korean adults aged 45 and older, underweight participants had higher levels of depressive symptoms compared to normal-weight participants. Moreover, there were no significant differences in depressive symptoms between normal weight, overweight, and obese participants (17). Finally, a study by Kim and colleagues (19) examined the association between weight and mental health status among a nationally representative sample of 2017 older adults aged 60 and older. The sample included 547 white, 814 black, 401 Hispanic, and 255 Asian older adults. Findings indicated that black and Asian older adults had better self-rated mental health at higher levels of BMI, whereas white and Hispanic older adults had poorer self-rated mental health at higher levels of BMI.

These studies document an association between obesity and mental health among older adults. However, the role of race and ethnicity in this association is not entirely clear. The race comparative approach employed in most of these studies has an underlying assumption that racial and ethnic groups are monolithic and, thus, can be compared to each other in the aggregate. However, the race comparative approach obscures important variation within racial and ethnic groups across a range of demographic, cognitive, social, and environmental factors that might help us better understand *how* obesity is associated with mental health in diverse populations.

## Empirical Investigations of Heterogeneity in Obesity and Mental Health Disorders

Studies of obesity and mental health indicate that the association between these conditions is inconsistent, with some studies reporting no association (6), others reporting a positive association (2,32), and still others reporting a negative association (7,8). However, many of these studies are limited to the extent that they consider heterogeneity in obesity and mental health disorders and how certain “types” or categories of these conditions are related to each other. Recent studies reporting improved cardiovascular prognosis (33) and reduced mortality among overweight and obese older adults as compared with leaner ones (34) suggest that the effects of obesity on health are not universal, but rather might be associated with different levels of disease risk. Findings from these studies point to a “healthy obese phenotype” which is defined as obesity in the absence of common metabolic complications of obesity (e.g., high blood pressure, high triglycerides, low high-density lipoprotein cholesterol, and elevated inflammatory markers). For example, Jokela and colleagues (35) examined the association between metabolic health and depressive symptoms using pooled data from eight cohort studies with more than 30,000 men and women aged 15–105 years old (mean age = 46.1). Findings indicated that both metabolically healthy and metabolically unhealthy obese participants had an increased risk of depressive symptoms. However, participants with a metabolically unhealthy obese profile had 23% higher odds of depressive symptoms compared to participants with a metabolically healthy obese profile (defined as no more than one metabolic risk factor).

In addition to examining variation in obesity profiles, it is also important to consider heterogeneity within mental health categories. Mental health disorders are not fixed and independent entities, but rather predict the future occurrence of the same disorder (homotypic continuity) and other disorders (heterotypic continuity) (36). Moreover, the course of mental illness varies between individuals and can be defined by transitions in and out of different stages over the course of the disease, including remission, relapse, recurrence, and persistence (37). There are also subcategories within a diagnosis which can pose different risks for obesity. A study by Chou and Yu (38) found that older adults with lifetime atypical depression (e.g., loss of appetite and initial or late insomnia) had an elevated risk of obesity compared to those with classic depression (e.g., hypersomnia and increased appetite), undifferentiated depression (e.g., did not meet criteria either for classic or for atypical depression), or no depression. Another notable study by Marijnissen and colleagues (39) examined the relationship between obesity and depressive symptoms taking into account different measures for obesity (BMI, waist circumference, and waist-to-hip ratio) and different depressive symptom clusters (cognitive-affective and somatic-affective symptoms) among 1,284 older adults. Findings indicated that depressive symptoms, overall, were associated with BMI, but only the somatic-affective symptom cluster was related to visceral obesity, which is a more specific measure of metabolic health than BMI.

Collectively, studies that consider population heterogeneity offer an approach to future studies that empirically capture the wide variation that exists within and across populations that are not solely defined by an obesity category or mental health diagnosis. Such investigations have the potential to reveal profiles, subpopulations, and/or risk types and can increase our understanding of the current body of evidence that demonstrates wide variation in outcomes across racial and ethnic groups that are regarded as inconsistent rather than a reflection of population heterogeneity. More systematic investigations are needed that explicitly examine obesity and mental health phenotypes among racial and ethnic minority older adults (40). We also need studies that go beyond describing obesity phenotypes in exclusively physiological terms. For example, what are the cognitive, behavioral, social, or environmental factors that define these phenotypes? What is the mental health profile of healthy phenotypes compared to unhealthy phenotypes? How many phenotypes exist and does the number of phenotypes vary by race or ethnicity? Additional studies are needed that examine a wide range of contextual factors, both independently and interactively, to determine an obesity–mental health typology among older racial and ethnic minority populations.

## Cognitive Mechanisms Link Race to Obesity and Mental Health

Perceived body weight is a mechanism that links obesity to mental health and potentially explains variation in this relationship across race and ethnicity. The idea of self-perceptions of weight is heavily rooted in cultural norms that define what is attractive and acceptable. Social differences in perceived body weight are especially relevant to the current discussion because culturally defined standards of beauty allow for variation in how individuals evaluate their body weight, which influences health and lifestyle decisions. Self-perception of body weight is also associated with mental health outcomes and, as such, is a common mechanism that links obesity to mental health. Negative body image and body dissatisfaction have been implicated as risk factors for various forms of psychopathology, including depression, anxiety, and eating disorders (41–43). Consequently, persons who are dissatisfied with their bodies are likely to be at great risk for mental health problems.

To date, most studies about perceptions of body weight have examined college students or young adults (44). Less is known about perceptions of weight in later life, perhaps because of the assumption that body issues diminish in importance among older adults. To the contrary, evidence suggests that concerns about aging and its effect on body weight and physical appearance are common in late life, particularly among older women (45,46). Studies also indicate that gender and race intersect to shape perceptions about body weight and size among older adults. In a study that compared black and white women aged 65 and older, Stevens and colleagues (47) documented that overweight black women were more satisfied with their weight, less likely to feel guilty after overeating, less likely to diet, and more likely to consider themselves attractive than overweight white women. They also found that, among women who were not overweight, white women were more likely than black women to describe themselves as overweight and report a lower ideal body weight.

Studies of perceived body image are important because of their potential for explaining the association between obesity and mental health outcomes, both independently of and in combination with relative body weight. These studies raise questions about using measures such as BMI to conceptualize obesity because it is a condition that is multidimensional. That is, obesity defined quantitatively, using BMI for example, might be useful for classifying individuals into weight categories that are generally associated with some level of health risk. However, obesity defined qualitatively (e.g., perceived body image) might be more useful for assessing the impact of weight on mental health status. Schieman and colleagues (48) used a sample of 1,164 older adults to examine gender, race, and socioeconomic status (SES) interactions on body image perceptions, including perceived weight versus actual BMI categories across gender–race groups. Findings indicated that black older adults were more likely than white older adults to underestimate their weight (i.e., describe themselves as appropriate weight when in fact they were classified in overweight or obese BMI categories). At higher levels of SES, black women were more likely than other race–gender groups to describe themselves as obese when they, in fact, had lower BMI than low-SES black women. Low-SES black women, however, had a higher BMI than high-SES black and white women, but they tended to perceive heavier body types as more attractive. In contrast, low-SES white women were more likely than the other race–gender groups to describe themselves as obese.

Studies that examine the impact of body image dissatisfaction (BID) on mental health among older adults are limited. However, a study by Gavin and colleagues (49) provides an empirical example of this relationship. These researchers examined the association between obesity, BID, and depression in a population-based sample of 4,660 middle-aged and older women. Obesity and BID increased the risk of depression among women with fewer years of education. For women with more years of education, obesity was not directly associated with depression, but increased their risk of depression through BID. The strength of the association between relative weight and body image among higher SES older women in this study is consistent with findings from Schieman and colleagues (48), Gavin and colleagues (50), and other studies showing that high-SES women report more dissatisfaction or concern with their bodies than low-SES women (with the exception of white women (48)). Findings from these studies identify body image perception as an important mediator in the relationship between obesity as defined by BMI and depression among certain segments of the population. Findings also provide insight into how core dimensions for stratification—race, gender, and SES—intersect to shape perceptions of body weight.

## Social and Behavioral Mechanisms Link Race to Obesity and Mental Health

One fairly recent hypothesis that attempts to explain the common mechanisms that link obesity and mental health is the Environmental Affordances (EA) model. Because the EA hypothesis considers a wide range of contextual factors, it does offer a fruitful approach for understanding *how* obesity and mental health are related and thus provides a roadmap for future studies. The EA hypothesis focuses on the social origins of health disparities and considers multiple pathways that connect race to mental health. These pathways include social factors as defined by exposure to psychosocial stress and behavioral factors as defined by coping behaviors (51). This hypothesis provides a framework for understanding the seemingly paradoxical association between obesity and mental health among some racial and ethnic minority groups—namely, higher rates of stress-related health conditions (e.g., obesity, cardiovascular disease, and type 2 diabetes) but lower rates of common forms of psychopathology (e.g., major depression and anxiety disorders) than whites. According to this hypothesis, empirical findings that indicate a lower prevalence of stress-related mental health disorders among blacks relative to whites, despite large disparities in stress-related health conditions in middle- and older age, are explained by unhealthy coping behaviors that suppress the effects of stress on mental health, but elevate the risk for obesity-related chronic health conditions.

Jackson and colleagues (18) conducted one of the first studies that tested the EA hypothesis by estimating the stress-buffering effects of unhealthy behaviors, measured by tobacco use, alcohol use, and obesity, on depression and chronic health conditions among African Americans and whites. Using data from two waves of the Americans’ Changing Lives Study and a sample of 872 African Americans and 1,906 whites, findings showed a moderating effect of unhealthy behaviors for both African Americans and whites, but in opposite ways. For African Americans, the relationship between stressful life events and depression was weakest for those who engaged in unhealthy behaviors compared to those who had not engaged in unhealthy behaviors. However, among whites, the opposite was found; the association between stressful life events and depression was stronger for those who engaged in more unhealthy behaviors than those who engaged in fewer unhealthy behaviors. Stressful life events increased the risk for chronic health conditions for both blacks and whites. However, unhealthy behaviors were positively associated with chronic health conditions for blacks but not for whites. Findings from this study suggest that unhealthy behaviors, such as smoking, overeating, and drinking alcohol, may alleviate the effects of stress on the mental health of blacks, but not whites. And it is these same behaviors that increase the risk for chronic health conditions, such as obesity, among blacks, but this was not the case for whites in this study.

Since this study, other researchers have tested the EA hypotheses using racially and ethnically diverse samples. Rodriquez and colleagues (20) used a sample of 6,479 middle-aged and older African Americans, Hispanics, and whites from the Health and Retirement Study to assess the role of unhealthy behaviors (i.e., current smoking, excessive and/or binge drinking, and obesity) in the relationship between chronic stress and depressive symptoms (20). Findings indicated that the interaction between chronic stress and unhealthy behaviors was significant for Hispanics, but not for African Americans or whites. For middle-aged and older Hispanics, the effect of chronic stress on depressive symptoms increased with each level of unhealthy behavior. Findings from this study also indicated that obesity increased the risk of depressive symptoms, but only for Hispanics.

One limitation of these studies that is worth noting is that the researchers used obesity as a proxy measure for poor diet and low physical activity. The existence of a metabolically healthy obesity phenotype challenges the assumption that obesity can be primarily defined by BMI, the types of food consumed, or the level of physical activity. Obesity is a dynamic condition that requires a wider range of measurements than what is currently used to capture it. An alternative approach to using obesity as a proxy measure for “diet” is to measure the type of food that is consumed. One study examined the relationship between food consumption, depressive symptoms, and body weight in a sample of 4,655 middle-aged women (52). The purpose of this study was to examine the extent to which the positive association between BMI and depressive symptoms was explained by specific food choices. Findings showed that women who consumed low-calorie foods (e.g., green salad, roast chicken, baked fish, and low-fat milk) reported fewer depressive symptoms and had a lower BMI than their counterparts. In contrast, women who consumed high-calorie sweet foods (e.g., cake, chocolate, cornbread, and soda) reported higher depressive symptoms but had a lower BMI than their counterparts. Finally, women who consumed high-calorie nonsweet foods (e.g., french fries, potato salad, pasta, chips, and cheese) reported fewer depressive symptoms but had a higher BMI compared to their counterparts. The principal finding from this study was that BMI and depressive symptoms appear to be associated with different food preferences, sweet foods in the case of lower BMI and higher depressive symptoms and nonsweet in the case of higher BMI and lower depressive symptoms. Both of these food types would be considered a “poor diet” but they have differential effects on weight and mental health outcomes.

Collectively, these studies suggest that some behaviors defined as unhealthy might serve a protective function against stress and, thus, explain variation in findings for obesity and mental health outcomes across older racial and ethnic minority groups. There is some indication from this body of evidence that stress coping with “unhealthy behaviors” is more protective for the mental health of middle-aged and older African Americans (18,19) than among older whites and Hispanics (18,20). However, more research is needed to clarify the role of race and ethnicity in these relationships. Findings of a preference for high-calorie sweets among older women with depressed mood provide additional insight by identifying food choice as a mechanism that links obesity to mental health disorders.

## Biological and Physiological Mechanisms Link Race to Obesity and Mental Health

Unhealthy behaviors such as smoking, drinking alcohol, and eating unhealthy foods may act along the same biological pathways that contribute to some mental health disorders (53,54). Studies that demonstrate the association between obesity and mental health disorders might, in fact, be demonstrating shared pathological mechanisms through the stress–response system rather than pathologies that presumably have different etiologies. The positive associations between stress, weight gain, obesity, and BMI are well documented (55,56). Furthermore, the connection between stress and metabolic dysfunction is stronger in individuals with higher BMI than in those with lower BMI (57), suggesting that stress elevates obesity risk especially in individuals with higher BMI. Moreover, unhealthy dietary habits, in particular, have been linked to alterations in the intestinal tract that could contribute to obesity and its metabolic and psychological complications (58). The higher prevalence of obesity in African Americans and Hispanics relative to whites has not been systematically explored along these lines.

Hypotheses such as the EA model are founded on the basis of the link between the environment and the stress response. However, studies that test the EA hypothesis do not directly test the biological stress–response system. Direct tests require studies that examine physiological responses to environmental factors. For example, gut microbiota is a mediating factor between the environment and health. Human gastrointestinal microbiota, also known as gut microbiota, are the microorganisms that live in the digestive tracts of humans which are extremely important to health and the progression of disease in older adults (59,60). Alteration of gut microbiota is affected by a variety of factors including exposure to stress, environment, diet, medications, stage of the lifecycle, and comorbid diseases. A number of diseases have been linked with alterations in the gut microbial community, including diabetes (61–63), obesity (64), and mental health disorders such as depression (65,66).

Research on the gut microbiota is currently revolutionizing our understanding of the links between the environment, obesity, and mental health. However, racial and ethnic diversity is generally underrepresented in current microbiota studies. Currently, there is only one study that examined differences in gut microbiota across race and ethnicity (67). Using a sample of healthy Asian-Pacific Islanders, whites, Hispanics, and African Americans, findings indicated that race and ethnicity consistently captured gut microbiota with a slightly stronger effect size than other variables such as BMI, age, and sex. Moreover, race and ethnicity were predictable from total gut microbiota differences. These findings suggest that microbiota differences by race and ethnicity have the potential to explain disparities in health and obesity and may be a common mechanism in the etiology of obesity and mental health. This line of inquiry is in its infancy and much more research is needed to understand the extent to which race and ethnicity can capture facets of biological variation. However, race and ethnicity are often used as a proxy for socioeconomic variation in population studies and to characterize health disparity incidence. Whether self-reported race and ethnicity are a useful proxy for biological variation will be determined, and perhaps challenged, in future studies. Nevertheless, findings from this study emphasize the importance of including racially and ethnically diverse samples in future studies to account for race and ethnic variation as a potential confounding factor in studies linking biological differences to disease.

## Conclusions

Understanding the link between obesity and mental health among racial and ethnic minority older adults has never been more important. The rise in obesity in the older adult population is a public health concern that will have a significant and potentially overwhelming impact on individuals, families, communities, and the health care system. While the prevalence of mental health disorders declines with age, the rates of obesity are rising the most among older adults in general and among racial and ethnic minority adults in particular. The potential for obesity to increase the risk for mental health problems in these populations is likely, but not very well understood. Racial and ethnic minority older adults, in particular black Americans and Hispanics, have the highest prevalence of overweight and obesity, but, overall, are reported to have relatively lower rates of mental health disorders compared to whites. However, a careful review of the studies that examine both obesity and mental health presents a more complex picture.

Our current understanding of the relationship between obesity and mental health among older racial and ethnic minority groups is limited to the extent that the body of research is generally pursued without consideration of race, ethnicity, or age, is primarily epidemiological with a focus on establishing prevalence rates, documents the link between obesity and mental health but falls short of identifying the mechanisms that link these conditions. Collectively, findings from this body of evidence indicate that the risk for mental health disorders in overweight and obese older adults will increase for some but not for others and that this might not be entirely determined by race or ethnicity. Because of population heterogeneity, it is more likely than not that there is a typology of obesity–mental health that is defined by a wide array of contextual factors, including race and ethnicity. Future studies are needed that examine a wider variety of causal factors that range from structural to physiological mechanisms and how these factors interact in diverse populations to affect obesity and mental health status.

Currently, the comparative approach that is common in many studies reviewed for this article obscures heterogeneity within populations and limits the ability to identify subpopulations of older adults with varying risk profiles. Thus, our greatest gap in knowledge is not regarding whether obesity and mental health are related, or whether race and ethnicity have some impact on the association between these conditions, but rather in how risk factors interact with one another to increase or decrease risk and, thus, who will be most at risk for poor outcomes. Future studies are needed that empirically identify risk and protective profiles using more rigorous methodological approaches, including latent class mixture models, longitudinal designs, and large racially and ethnically diverse samples.

Findings from the studies reviewed, collectively, have the potential to move the field forward and bring us closer to understanding common etiology between obesity and mental health, and to discovering phenotypes that pose the highest risk for health complications. Existing studies have been very useful for identifying structural, social, behavioral, and cognitive factors that explain variation in outcomes across groups—whether these groups are distinguished by obesity class, mental health status, race, or ethnicity. The idea that gut microbiota can be determined by race and ethnicity is a groundbreaking discovery with significant implications for understanding how structural and social determinants of health “get under the skin” and lead to the tremendous variation observed in obesity and mental health outcomes in older minority adults. Future studies should continue to examine these contextual factors interactively, but should prioritize studies that more explicitly examine how racism structures environmental and social exposures, health behaviors, access to health care and health information, and mental health service utilization.

The field has advanced in the identification of important risk factors and causal mechanisms linking obesity and mental health disorders, but still has not reached the stage where we can determine the level of risk—high or low—for comorbid obesity and mental health disorders among older minority populations. Some gaps can be filled by improving the methodology with better representation of older racial and ethnic minorities in studies, stratified data analysis, longitudinal studies to capture the course of the disease, and the use of more comprehensive measures that conceptualize the dynamic nature of obesity and mental health disorders. In other cases, an interdisciplinary approach is needed to capture the array of causal factors that have yet to be considered jointly to better understand the pathways whereby structural factors get under the skin. In the end, a reasonable goal of this research is to be able to predict which older adult is more likely to suffer from comorbid obesity and mental health problems and to be able to prevent, delay, or treat them to improve their quality of life.
